# Supplementary material for: Long-term persistence of infectious Zika virus: Inflammation and behavioral sequela in mice
Source: PLoS Pathog. 2020 Dec 10;16(12):e1008689. doi: 10.1371/journal.ppat.1008689 (PMC7728251; doi:10.1371/journal.ppat.1008689)
Supplement: S1 Table — Spearman’s rank correlation were used to compare brain characteristics as measure during MRI volumetric analysis and the indicated behavioral parameters. Statistically significant correlations are indicated with blue, underlined values. (PDF) [file ppat.1008689.s001.pdf]

	MRI Score			Correlation of MRI and Behavioral Score																	
	Mean (SD)			Weight	Rotarod	OFT				EPM				NOR				SI			
						Total		50%		Total		Open		Interaction		Proximity		%	Ratio		
MRI Measurements	Uninfected	Infected	p (infected vs unifected)	Weight (g)	Latency to Fall (s)	Distance (m)	Velocity (mm/s)	Distance (m)	Duration (s)	Distance traveled (m)	Time (s)	Distance traveled (m)	Time (s)	% Time exploring novel object	Time novel object:Time familiar object	% Time exploring novel object	Time novel object:Time familiar object	Stranger	Social:Non	Stranger: Empty	
Weight (g)	26.2 (1.3)	26.9 (1.4)	NS	1	-0.14	-0.26	0.31	-0.27	-0.12	-0.04	0.03	-0.29	-0.27	0.38	0.24	0.1	0.1	0.1	0.24	0.12	
Total Brain Volume (mm^3)	468.8 (1.1)	428.3 (1.1)	***	0.18	0.47	-0.6	-0.2	-0.13	0.38	-0.29	-0.16	-0.52	-0.63	0.24	0.35	0.07	0.07	0.19	0.02	0.27	
Brain Vol (parenchyma) (mm^3)	447.3 (1.1)	397.1 (1.1)	***	0.09	0.53	-0.59	-0.23	-0.13	0.38	-0.31	-0.16	-0.54	-0.64	0.18	0.27	0.03	0.03	0.21	0.04	0.3	
Fluid Vol (mm^3)	19.7 (1.5)	28.8 (1.3)	**	0.07	-0.38	0.23	0.13	0.11	-0.12	0.28	-0.09	0.37	0.28	0.04	0.17	0.06	0.06	-0.19	-0.3	-0.25	
% Fluid volume	4.2% (1.5%)	6.7% (1.3%)	***	0	-0.46	0.38	0.14	0.12	-0.22	0.34	-0.02	0.46	0.4	-0.04	0.04	0.04	0.04	-0.19	-0.29	-0.27	
Cerebellum (mm^3)	53.7 (1.1)	33.7 (1.6)	***	0.2	0.44	-0.64	-0.22	-0.22	0.3	-0.18	-0.44	-0.55	-0.63	0.37	0.45	0.27	0.27	0.09	-0.05	0.17	
