# Supplementary material for: Long-term persistence of infectious Zika virus: Inflammation and behavioral sequela in mice
Source: PLoS Pathog. 2020 Dec 10;16(12):e1008689. doi: 10.1371/journal.ppat.1008689 (PMC7728251; doi:10.1371/journal.ppat.1008689)
Supplement: S2 Table — Spearman’s rank correlations were used to compare immune gene expression and the indicated behavioral parameters. Statistically significant correlations are indicated with blue, underlined values. (PDF) [file ppat.1008689.s002.pdf]

TABLE 2: mRNA expression in cerebellum and behavioral testing scores		Gene Expression Score			Correlation of Gene Expression and Behavioral Score																
		GMean (GSD)			Weight	Rotarod	OFT				EPM				NOR				SI		
							Total		50%		Total		Open		Interaction		Proximity		%	Ratio	
	Gene Expression	Uninfected	Infected	p (infected vs unifected)	Weight (g)	Latency to Fall (s)	Distance (m)	Velocity (mm/s)	Distance (m)	Duration (s)	Distance traveled (m)	Time (s)	Distance traveled (m)	Time (s)	% Time exploring novel object	Time novel object:Time familiar object	% Time exploring novel object	Time novel object:Time familiar object	Stranger	Social:Non	Stranger: Empty
Antigen Presentation	B2m	1.3 (2.1)	4.2 (1.5)	**	-0.39	-0.49	0.60	0.13	0.21	-0.29	0.17	0.21	0.56	0.58	-0.14	-0.22	-0.01	-0.01	-0.04	0.00	-0.12
	Cd40	1.3 (2.6)	4.3 (2.4)	*	-0.33	-0.24	0.57	0.22	0.24	-0.24	0.30	0.13	0.50	0.50	-0.31	-0.40	-0.16	-0.16	0.07	0.04	-0.08
	Cd68	1.4 (3.3)	6.8 (2.3)	**	-0.34	-0.50	0.63	0.18	0.25	-0.24	0.10	0.31	0.53	0.58	-0.15	-0.23	-0.03	-0.03	0.02	0.04	-0.04
	Cd80	1.3 (2.6)	3.2 (1.8)	*	-0.35	-0.51	0.67	0.12	0.24	-0.35	0.28	0.08	0.46	0.51	-0.12	-0.08	0.16	0.16	-0.16	-0.02	-0.18
	Cd86	1.3 (2.5)	4.2 (1.7)	**	-0.31	-0.57	0.64	0.25	0.28	-0.32	0.23	0.19	0.57	0.56	-0.19	-0.28	-0.12	-0.12	-0.17	-0.10	-0.23
	H2-Eb1	1.5 (4)	12.3 (3.4)	**	-0.35	-0.46	0.71	0.25	0.37	-0.21	0.32	0.23	0.59	0.61	-0.27	-0.30	-0.06	-0.06	-0.06	0.03	-0.19
Chemokines & Receptors	Ccl19	1.2 (2.1)	3.4 (1.7)	**	-0.32	-0.52	0.71	0.21	0.19	-0.39	0.26	0.32	0.57	0.60	-0.21	-0.28	0.00	0.00	-0.15	-0.09	-0.25
	Ccl2	1.4 (3.9)	4.6 (2.1)	**	-0.27	-0.53	0.70	0.21	0.21	-0.38	0.19	0.34	0.51	0.51	-0.35	-0.34	-0.06	-0.06	-0.11	-0.08	-0.25
	Ccl3	1.7 (11)	137.4 (2.1)	**	-0.26	-0.29	0.58	0.31	0.10	-0.29	0.03	0.41	0.51	0.52	-0.29	-0.41	-0.26	-0.26	0.16	0.13	-0.01
	Ccl5	1.6 (4.4)	14.1 (3.2)	**	-0.36	-0.50	0.66	0.18	0.28	-0.23	0.14	0.28	0.58	0.62	-0.15	-0.23	0.04	0.04	-0.03	-0.01	-0.09
	Ccr2	1.3 (2.2)	4 (1.9)	**	-0.35	-0.53	0.70	0.24	0.23	-0.42	0.29	0.20	0.60	0.60	-0.19	-0.24	-0.11	-0.11	-0.16	-0.06	-0.27
	Ccr4	1.3 (2.4)	1.1 (2.2)	NS	-0.08	0.22	-0.12	-0.04	-0.19	0.07	-0.30	0.06	-0.09	-0.06	-0.02	-0.12	0.00	0.00	0.44	0.18	0.47
	Ccr7	1.4 (3.6)	3.1 (3.8)	NS	-0.26	-0.47	0.29	-0.01	0.15	-0.01	-0.14	0.20	0.06	0.21	-0.11	-0.16	-0.05	-0.05	0.24	0.22	0.41
	Cxcl10	1.5 (3.8)	7.8 (2.7)	**	-0.37	-0.43	0.65	0.15	0.20	-0.31	0.11	0.32	0.53	0.59	-0.22	-0.31	0.00	0.00	-0.01	-0.02	-0.15
	Cxcl11	1.6 (5.1)	17.9 (2.7)	**	-0.31	-0.61	0.69	0.21	0.31	-0.32	0.24	0.27	0.62	0.65	-0.06	-0.09	0.04	0.04	-0.28	-0.09	-0.33
	Cxcr3	1.7 (7.1)	23 (4.8)	**	-0.39	-0.35	0.59	0.12	0.16	-0.27	0.16	0.28	0.55	0.61	-0.22	-0.31	0.00	0.00	-0.03	-0.02	-0.13
Cytokines	Gzmb	1.3 (3.4)	13.5 (2.2)	**	-0.03	-0.30	0.59	0.44	0.11	-0.36	0.00	0.26	0.47	0.52	-0.24	-0.38	-0.15	-0.15	0.14	0.24	-0.01
	Ifng	1.3 (2.8)	4.8 (2.2)	**	-0.12	-0.58	0.57	0.30	0.34	-0.23	0.25	0.19	0.53	0.48	-0.12	-0.17	-0.10	-0.10	-0.24	-0.13	-0.27
	Il10	1.3 (2.3)	3.1 (2.5)	*	-0.21	-0.43	0.46	0.25	0.17	-0.24	0.12	0.06	0.40	0.42	-0.10	-0.13	-0.20	-0.20	-0.10	0.04	-0.12
	Il13	1.2 (1.9)	1.4 (2.5)	NS	0.14	0.00	0.11	0.52	0.08	-0.15	0.18	0.09	0.25	0.09	0.06	-0.03	-0.32	-0.32	-0.18	0.00	-0.24
	Il2	1.2 (1.9)	1.8 (2.2)	NS	-0.09	-0.18	0.36	0.33	-0.10	-0.43	0.02	0.22	0.40	0.48	-0.01	-0.15	0.01	0.01	0.02	0.18	0.01
	Il3	1 (1.3)	1.2 (1.9)	NS	0.15	0.12	0.04	0.46	-0.14	-0.18	0.04	0.26	0.28	0.25	-0.08	-0.18	-0.26	-0.26	-0.03	0.18	-0.07
	Il5	0.9 (2.1)	4.4 (3.1)	**	0.20	-0.14	0.34	0.55	0.18	-0.21	0.17	0.29	0.45	0.40	-0.16	-0.31	-0.28	-0.28	0.08	0.26	-0.07
	Il7	1.5 (4.3)	18.3 (2.2)	**	-0.28	-0.55	0.74	0.23	0.21	-0.47	0.23	0.20	0.59	0.62	-0.15	-0.21	-0.01	-0.01	-0.35	-0.18	-0.45
	Prf1	1.4 (3.8)	3.8 (3.4)	NS	-0.22	-0.34	0.59	0.35	0.32	-0.25	0.44	0.15	0.68	0.69	-0.15	-0.16	0.02	0.02	-0.26	0.04	-0.38
	Tgfb1	1.3 (3)	2.1 (2.5)	NS	-0.51	-0.30	0.47	-0.17	0.17	-0.20	0.15	0.09	0.32	0.37	-0.34	-0.33	0.15	0.15	0.08	0.19	-0.08
Inflammation	Agtr2	1.2 (2.6)	2.3 (1.9)	NS	-0.45	-0.47	0.57	0.04	0.42	-0.19	0.32	0.14	0.50	0.46	-0.29	-0.24	-0.05	-0.05	-0.47	-0.29	-0.67
	C3	2 (10.7)	109.7 (3.5)	**	-0.36	-0.52	0.67	0.20	0.25	-0.26	0.12	0.35	0.57	0.63	-0.21	-0.30	-0.05	-0.05	0.02	0.07	-0.07
	Col4a5	1.1 (1.6)	2.1 (1.4)	**	-0.44	-0.43	0.63	0.21	0.16	-0.31	0.24	0.25	0.60	0.64	-0.25	-0.25	-0.11	-0.11	-0.08	0.03	-0.21
	Csf1	1.2 (1.6)	2.1 (1.3)	**	-0.33	-0.62	0.69	0.22	0.39	-0.26	0.33	0.12	0.59	0.57	-0.16	-0.15	-0.06	-0.06	-0.40	-0.26	-0.46
	Csf2	1.2 (2.6)	1.4 (3)	NS	-0.14	0.21	0.24	0.29	0.07	-0.05	0.00	-0.19	0.14	0.12	0.02	-0.09	-0.04	-0.04	0.19	0.10	0.19
	Csf3	1 (2.1)	0.7 (2.3)	NS	0.12	0.48	-0.56	-0.30	-0.46	0.14	-0.31	0.07	-0.36	-0.40	0.10	-0.02	0.03	0.03	0.28	0.01	0.31
	Cyp1a2	1.1 (4.1)	0.8 (4.1)	NS	-0.38	0.00	-0.10	-0.27	-0.06	0.00	-0.16	0.05	-0.03	-0.07	-0.03	-0.13	-0.06	-0.06	0.09	-0.10	0.10
	Cyp7a1	1 (2)	0.9 (2)	NS	-0.08	-0.02	0.00	0.11	-0.12	-0.19	-0.05	0.12	0.21	0.08	0.03	-0.04	0.04	0.04	-0.30	-0.41	-0.30
	Fn1	1.1 (1.4)	1.8 (1.5)	**	-0.46	-0.37	0.56	0.07	0.16	-0.30	0.36	0.16	0.62	0.65	-0.22	-0.28	0.08	0.08	-0.07	-0.01	-0.16
	Gusb	1.1 (1.5)	1.8 (1.3)	**	-0.35	-0.48	0.57	0.25	0.24	-0.28	0.30	0.22	0.63	0.58	-0.18	-0.18	-0.13	-0.13	-0.17	-0.07	-0.28
	Hmox1	1.1 (1.6)	2.3 (1.4)	**	-0.27	-0.56	0.63	0.30	0.25	-0.32	0.24	0.27	0.62	0.60	-0.13	-0.24	-0.11	-0.11	-0.12	-0.04	-0.22
	Il12a	1.1 (2.1)	1 (2.8)	NS	0.11	-0.30	-0.04	0.01	0.01	0.01	0.01	-0.05	-0.06	-0.23	-0.07	0.00	-0.06	-0.06	-0.40	-0.63	-0.31
	Il12b	1.3 (3)	3.1 (3.2)	NS	-0.28	0.01	0.38	0.23	-0.07	-0.39	0.10	0.08	0.34	0.40	-0.27	-0.37	-0.29	-0.29	0.13	0.16	-0.05
	Il18	1.2 (1.8)	2 (1.6)	**	-0.34	-0.60	0.66	0.20	0.41	-0.27	0.38	0.13	0.64	0.57	-0.19	-0.20	-0.06	-0.06	-0.29	-0.15	-0.43
	Il1a	1.3 (2.7)	3.7 (1.7)	**	-0.25	-0.58	0.72	0.27	0.28	-0.41	0.22	0.35	0.64	0.64	-0.14	-0.22	-0.07	-0.07	-0.28	-0.07	-0.39
	Il1b	1.2 (2)	1.3 (2.4)	NS	-0.39	-0.22	0.23	-0.01	0.29	0.12	0.17	-0.05	0.17	0.06	-0.33	-0.25	-0.39	-0.39	0.18	-0.07	0.06
	Il6	1.2 (2.9)	2 (2.5)	NS	0.06	-0.13	0.18	0.37	0.37	0.13	0.28	-0.05	0.32	0.19	0.07	0.07	-0.23	-0.23	0.01	0.24	-0.15
	Lrp2	1.1 (1.8)	2.3 (1.7)	**	-0.28	-0.32	0.65	0.24	0.31	-0.27	0.40	0.30	0.69	0.63	-0.23	-0.29	0.04	0.04	-0.13	-0.05	-0.28
	Nos2	1.1 (1.6)	1.4 (1.6)	NS	-0.08	-0.47	0.43	0.19	0.15	-0.39	0.20	0.00	0.34	0.33	0.15	0.11	0.15	0.15	-0.27	-0.02	-0.25
	Ptgs2	1.3 (2.4)	2.7 (2.1)	*	-0.25	-0.49	0.51	0.06	0.20	-0.21	0.13	0.33	0.44	0.41	-0.13	-0.16	0.03	0.03	0.01	-0.16	-0.14
	Ptprc	1.3 (2.7)	5.2 (1.9)	**	-0.34	-0.52	0.61	0.20	0.24	-0.28	0.22	0.28	0.59	0.59	-0.19	-0.24	-0.10	-0.10	-0.04	-0.03	-0.16
	Sele	0.9 (3.3)	0.9 (3.6)	NS	-0.13	0.30	-0.22	-0.17	-0.02	0.32	-0.28	0.07	-0.15	-0.19	-0.04	-0.07	0.03	0.03	0.13	-0.13	0.20
	Selp	1 (3.2)	1.9 (2.8)	NS	-0.17	-0.18	0.15	0.03	-0.23	-0.27	-0.32	0.16	0.28	0.30	-0.30	-0.32	-0.03	-0.03	-0.17	-0.35	-0.12
	Tnf	1.4 (4.2)	9 (2.9)	**	-0.37	-0.25	0.49	0.13	0.04	-0.33	0.04	0.14	0.44	0.49	-0.20	-0.29	-0.13	-0.13	0.13	0.08	0.01
	Vcam1	1.2 (1.9)	3 (1.7)	**	-0.39	-0.53	0.75	0.24	0.37	-0.26	0.27	0.21	0.60	0.61	-0.27	-0.26	-0.10	-0.10	-0.25	-0.16	-0.34
Lymphocyte	Cd19	1.5 (4.1)	6 (5.1)	NS	-0.31	-0.40	0.50	0.01	0.21	-0.24	0.35	0.28	0.48	0.44	-0.05	-0.06	0.14	0.14	-0.20	-0.20	-0.28
	Cd28	1.5 (4.4)	6.5 (3)	**	-0.37	-0.46	0.65	0.20	0.47	-0.05	0.27	0.06	0.54	0.57	-0.09	-0.07	0.08	0.08	-0.29	-0.18	-0.22
	Cd34	1.1 (1.3)	1.5 (1.4)	NS	-0.39	-0.21	0.51	0.07	0.14	-0.33	0.30	-0.02	0.48	0.43	-0.21	-0.33	0.01	0.01	-0.16	-0.16	-0.29
	Cd38	1.2 (1.9)	2.7 (1.6)	**	-0.34	-0.53	0.70	0.24	0.27	-0.35	0.26	0.30	0.61	0.66	-0.18	-0.29	0.01	0.01	-0.09	0.06	-0.20
	Cd3e	1.5 (4.3)	22.9 (2.3)	**	-0.32	-0.48	0.65	0.27	0.32	-0.20	0.18	0.21	0.61	0.64	-0.14	-0.23	-0.02	-0.02	-0.03	0.02	-0.09
	Cd4	1.3 (3.1)	6.1 (2.6)	**	-0.09	-0.71	0.62	0.33	0.24	-0.35	0.26	0.19	0.45	0.48	0.04	0.02	0.03	0.03	-0.19	-0.03	-0.16
	Cd40lg	1.3 (2.2)	2.6 (2.4)	*	0.14	-0.59	0.37	0.41	0.23	-0.22	0.10	0.12	0.40	0.33	0.19	0.17	-0.07	-0.07	-0.36	-0.12	-0.36
	Cd8a	1.6 (4.6)	31.1 (2.4)	**	-0.37	-0.51	0.65	0.15	0.23	-0.30	0.14	0.27	0.57	0.63	-0.11	-0.20	0.06	0.06	-0.13	-0.01	-0.13
	Ctla4	1.6 (4.3)	7.4 (6.3)	*	-0.42	-0.28	0.45	0.10	0.20	-0.21	0.34	0.06	0.53	0.53	0.01	-0.07	0.04	0.04	-0.12	0.02	-0.17
	Fas	1.2 (2.1)	2.2 (1.6)	**	-0.37	-0.47	0.63	0.15	0.25	-0.29	0.21	0.34	0.54	0.56	-0.25	-0.35	-0.01	-0.01	-0.03	0.02	-0.11
	Fasl	1.2 (1.8)	1.8 (1.8)	NS	-0.19	-0.34	0.31	0.04	0.32	0.03	0.19	0.06	0.35	0.31	-0.09	-0.19	-0.03	-0.03	0.20	0.17	0.07
	GITR	1.5 (3.8)	16.9 (2.4)	**	-0.32	-0.54	0.69	0.38	0.26	-0.32	0.23	0.29	0.68	0.65	-0.26	-0.29	-0.21	-0.21	-0.24	-0.22	-0.31
	Icos	1.4 (4.1)	8.4 (4.7)	*	-0.13	-0.74	0.46	0.17	0.41	-0.12	0.21	0.12	0.52	0.41	0.10	0.10	-0.01	-0.01	-0.39	-0.26	-0.40
	Il2ra	1.3 (2.9)	2.6 (2.8)	NS	-0.31	-0.46	0.48	0.21	0.38	-0.13	0.24	0.14	0.47	0.44	-0.11	-0.15	-0.22	-0.22	-0.30	-0.22	-0.39
	Stat1	1.4 (3.2)	3.8 (2.5)	**	-0.27	-0.45	0.77	0.20	0.33	-0.37	0.18	0.16	0.55	0.58	-0.26	-0.29	0.05	0.05	-0.24	-0.01	-0.43
	Stat4	1.4 (4.3)	11.1 (3)	**	-0.47	-0.46	0.63	0.13	0.28	-0.29	0.28	0.15	0.65	0.66	-0.16	-0.16	0.01	0.01	-0.34	-0.19	-0.39
	Stat6	1.1 (1.4)	1.7 (1.4)	**	-0.36	-0.46	0.52	0.19	0.23	-0.24	0.23	0.22	0.62	0.60	-0.17	-0.24	-0.17	-0.17	0.04	0.09	-0.11
	Tbx21	1.5 (4.2)	6.3 (2.9)	**	-0.35	-0.62	0.66	0.19	0.31	-0.30	0.22	0.31	0.58	0.54	-0.14	-0.11	-0.09	-0.09	-0.43	-0.41	-0.48
