# Supplementary figures and images for: Long-term persistence of infectious Zika virus: Inflammation and behavioral sequela in mice
Source: PLoS Pathog. 2020 Dec 10;16(12):e1008689. doi: 10.1371/journal.ppat.1008689 (PMC7728251; doi:10.1371/journal.ppat.1008689)

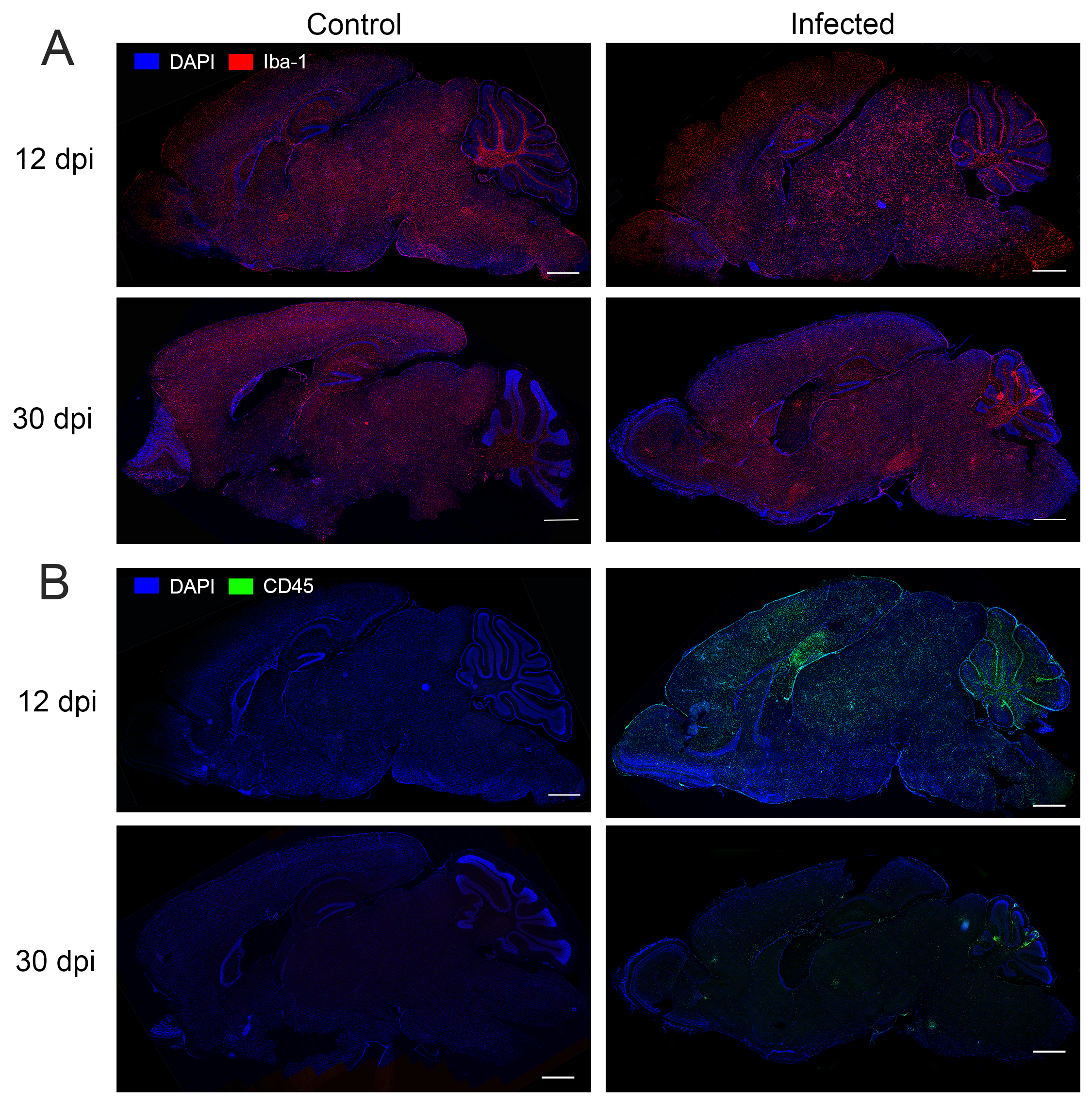

Supplement: S1 Fig — Sagittal sections from control and ZIKV-infected brains at 12 and 30 dpi. A. Sections stained with Iba-1 (red) to mark microglia. Note activation of microglia through the brain at 12 dpi is significantly reduced by 30 dpi in the cerebral cortex, hippocampus and thalamic regions. More focal microglia activation is found in the cerebellum at 30 dpi. B. Sections stained with CD45 (green) to mark infiltrating immune cells. Images representative of 3–5 mice. (TIF) [file ppat.1008689.s003.tif]

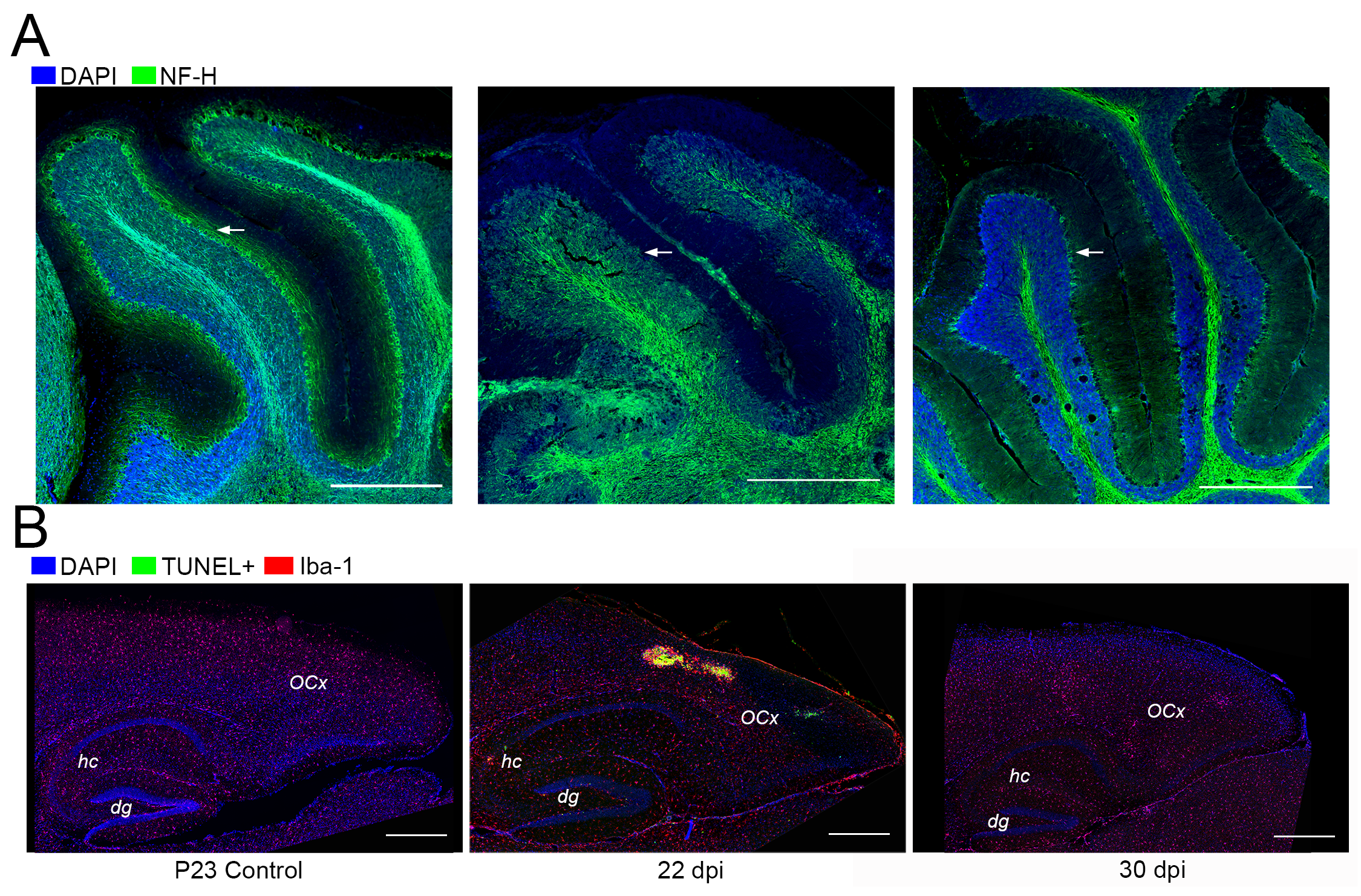

Supplement: S2 Fig — A. Cerebellum of control and ZIKV infected mice stained with DAPI (Blue) and Neurofilament staining (green). Arrow indicates Purkinje cell layer. B. Regions in the cerebral cortex and hippocampus stain positively for apoptosis (TUNEL, green) and the activation of microglia (Iba-1+, red) at 22 dpi. These lesions are resolved by 30 dpi. dg = dentate gyrus, hc = hippocampus, OCx = Occipital cortex. Scale bars (A&B) = 500 μm. (TIF) [file ppat.1008689.s004.tif]

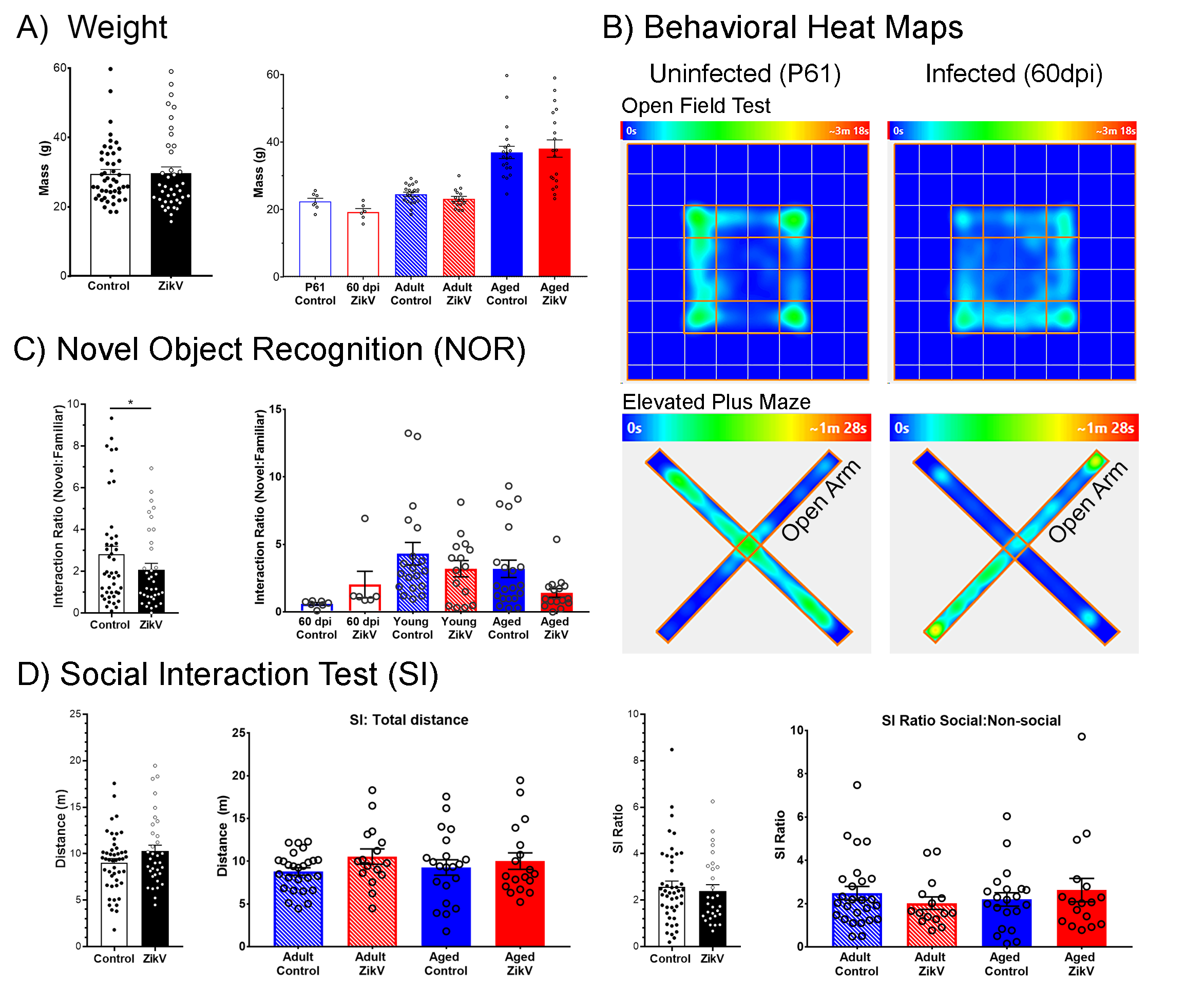

Supplement: S3 Fig — A. Weight of the animals tested in behavioural studies. B. Representative examples of heat tracking maps for 60 dpi mice recorded in the open field and Elevated Plus Maze tests (representative of 6–7 mice/group). The open arm of the maze is labelled. The opposite arm is closed. C. Novel Object recognition (NOR). Bars show interaction ratio (Novel:Familiar) as the time spent with new vs familiar objects D. Social Interaction Test. Data indicates total movement in the maze and Sociability index (SI, Ratio of Social:Non-social with mice in the maze). Statistical analysis between infected and uninfected groups: T test. * P<0.05. (TIF) [file ppat.1008689.s005.tif]

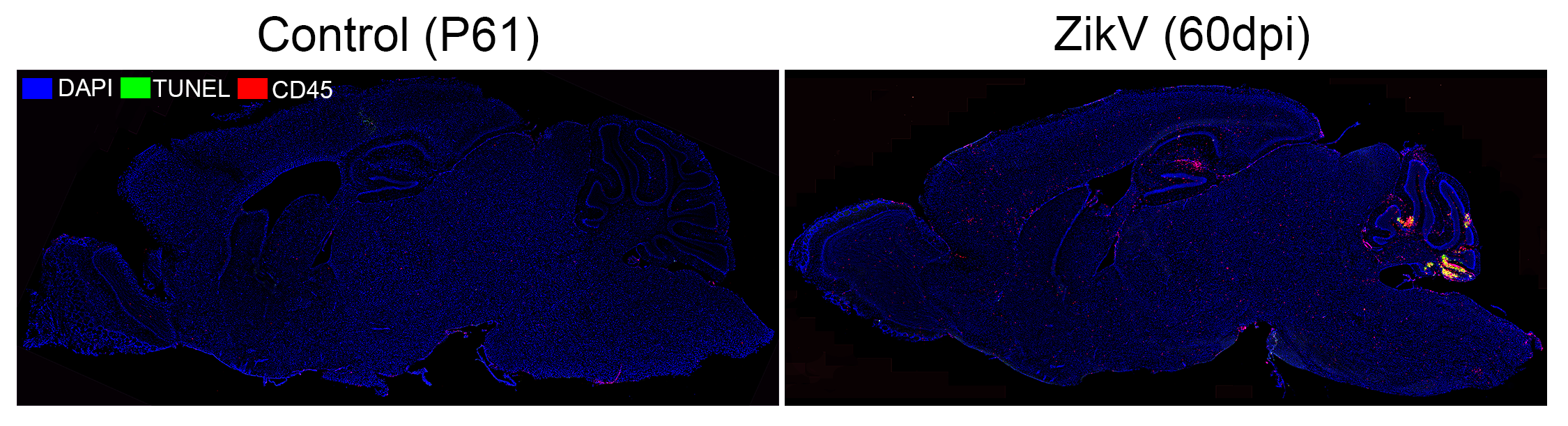

Supplement: S4 Fig — Sagittal sections stained for apoptotic (TUNEL+, green), and immune infiltrating (CD45+, red) cells. Evidence of CD45+ cells and apoptosis in hippocampus and cerebellum convalescent animals at 60 dpi. Image representative of 6 animals. (TIF) [file ppat.1008689.s006.tif]

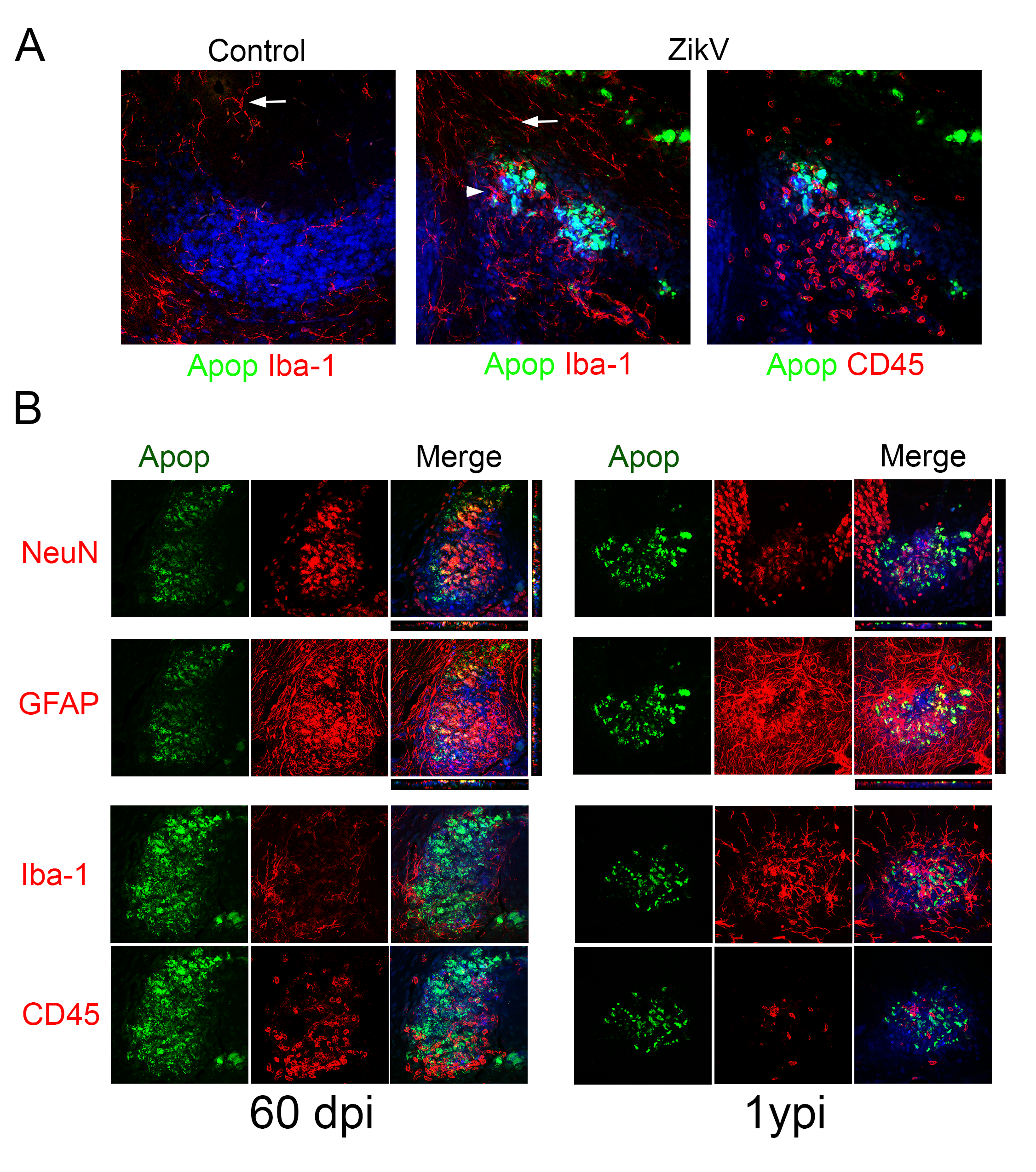

Supplement: S5 Fig — A. Confocal image of ZIKV+ foci in cerebellum of 1 ypi ZIKV-infected mice, shows position of TUNEL+ cells (apoptosis, green) relative to Iba+ microglia (red) and infiltrating CD45+ cells (red). Note that microglia proximal to apoptotic lesions shows hypertrophy of the nuclei and short processes consistent with activation (arrow head), while those distant to the lesion show small nuclei and fine processes (arrow). CD45+ infiltrating lymphocytes are also proximal to apoptotic foci. B. Individual channels for: Apoptosis (Apop, green), astrocytes (GFAP, red), microglia (Iba-1, red) and infiltrating lymphocytes (CD45+, red) and merged images with orthogonal views, for apoptotic lesions also shown in Fig 5. (TIF) [file ppat.1008689.s007.tif]

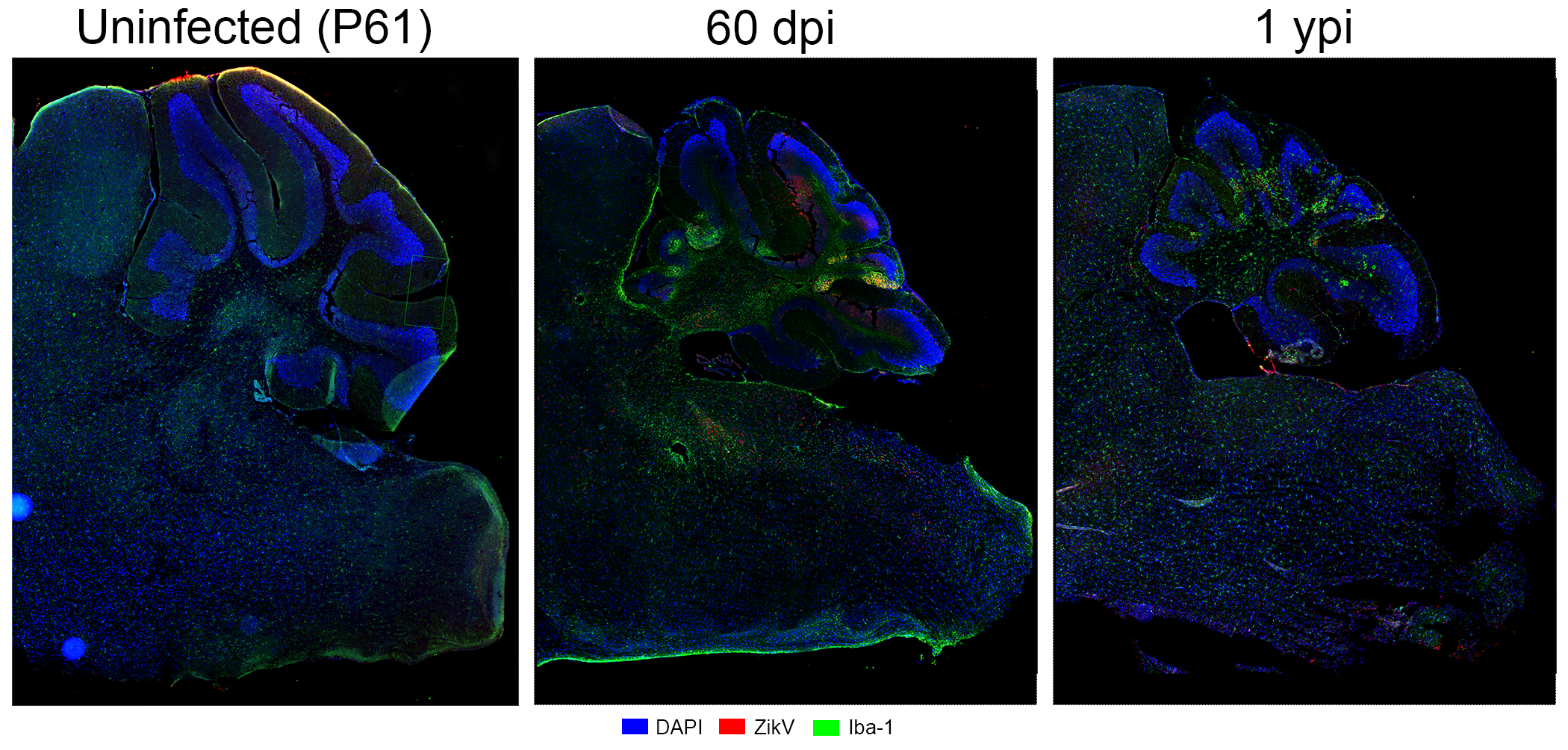

Supplement: S6 Fig — Images of the cerebellum and brain stain, stained for Iba-1 (green), Zikv, using signal amplification (red) and DAPI (Blue) indicate specific regions of ZIKV infection, associated with foci activated microglia, as indicated in Fig 7. (TIF) [file ppat.1008689.s008.tif]

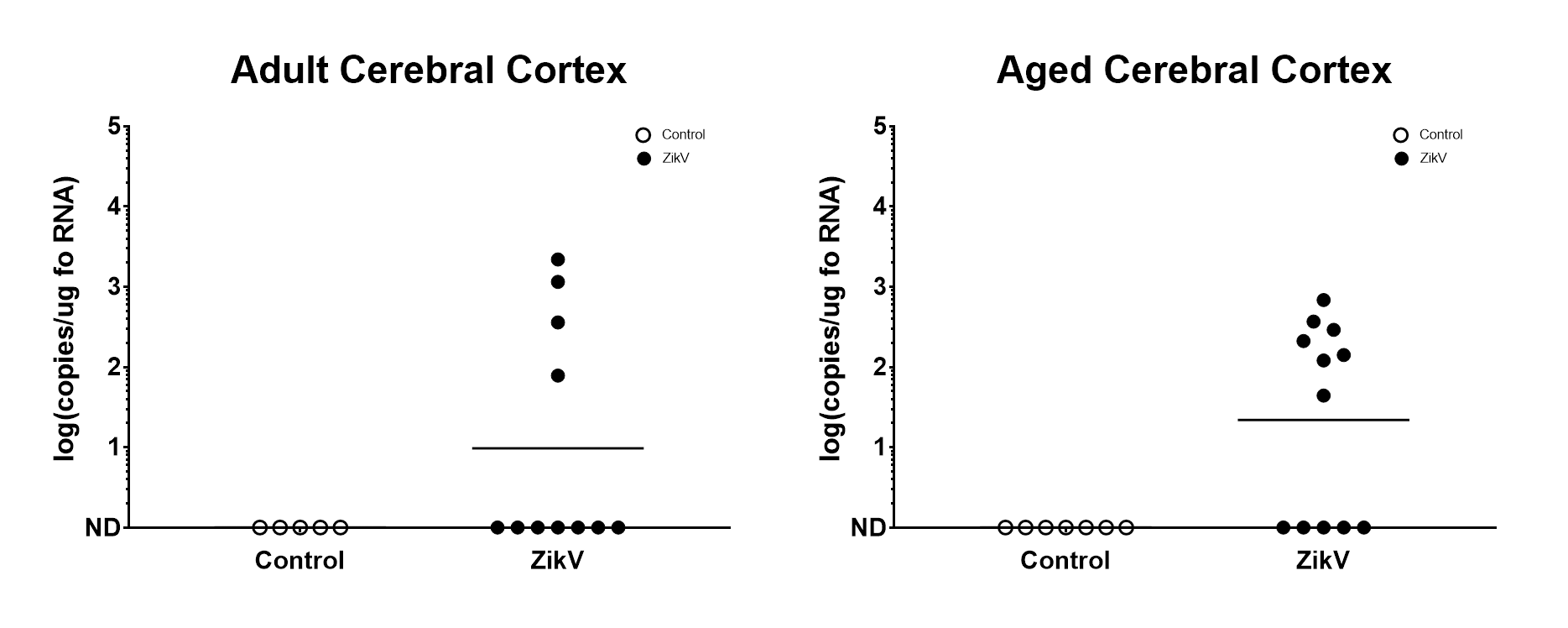

Supplement: S7 Fig — Single reaction taqman RT-qPCR for viral RNA performed on total RNA isolated from the cerebral cortex of ZIKV-infected or age-matched control animals. A sub-population of animals demonstrated low levels of viral RNA in the cerebral cortex. (TIF) [file ppat.1008689.s009.tif]
